# Parkinson's image detection and classification based on deep learning

**DOI:** 10.1186/s12880-024-01364-8

**Published:** 2024-07-25

**Authors:** Hui Li, Zixuan Yang, Weimin Qi, Xinchen Yu, Jiaying Wu, Haining Li

**Affiliations:** 1 Department of Computer Engineering, Jiangsuiangsu Ocean University, Lianyungang, 222005 China; 2 Department of Neurology, General Hospital of Ningxia Medical, Ningxia, 750004 China

**Keywords:** Parkinson’s disease, YOLOv5, Coordinate Attention, Dynamic full dimensional convolution, Decoupling joint

## Abstract

**Objective:**

There are two major issues in the MRI image diagnosis task for Parkinson's disease. Firstly, there are slight differences in MRI images between healthy individuals and Parkinson's patients, and the medical field has not yet established precise lesion localization standards, which poses a huge challenge for the effective prediction of Parkinson's disease through MRI images. Secondly, the early diagnosis of Parkinson's disease traditionally relies on the subjective judgment of doctors, which leads to insufficient accuracy and consistency. This article proposes an improved YOLOv5 detection algorithm based on deep learning for predicting and classifying Parkinson's images.

**Methods:**

This article improves the YOLOv5s network as the basic framework. Firstly, the CA attention mechanism was introduced to enable the model to dynamically adjust attention based on local features of the image, significantly enhancing the sensitivity of the model to PD related small pathological features; Secondly, replace the dynamic full dimensional convolution module to optimize the multi-level extraction of image features; Finally, the coupling head strategy is adopted to improve the execution efficiency of classification and localization tasks separately.

**Results:**

We validated the effectiveness of the proposed method using a dataset of 582 MRI images from 108 patients. The results show that the proposed method achieves 0.961, 0.974, and 0.986 in Precision, Recall, and mAP, respectively, and the experimental results are superior to other algorithms.

**Conslusion:**

The improved model has achieved high accuracy and detection accuracy, and can accurately detect and recognize complex Parkinson's MRI images.

**Significance:**

This algorithm has shown good performance in the early diagnosis of Parkinson's disease and can provide clinical assistance for doctors in early diagnosis. It compensates for the limitations of traditional methods.

## Introduction

Parkinson's disease (PD) is a neurodegenerative disease that mainly affects the central nervous system, causing motor dysfunction and other multi-system symptoms [1]. Parkinson's disease is usually caused by a variety of factors, including genetics, environment, and lifestyle. Its incidence is increasing year by year and has become an important public health issue around the world. The clinical manifestations of Parkinson's disease mainly contain resting tremors, muscle stiffness, slow movement, and postural balance disorder [2]. As the disease progresses, patients may experience symptoms like language, cognitive, and emotional disorders, seriously affecting their quality of life [3]. Currently, although medications and physical therapy can help relieve the symptoms of Parkinson's disease, there is no cure.Due to the small and inconspicuous lesion areas in the early stages of Parkinson's disease on MRI images, traditional diagnostic methods make it difficult to achieve accurate and efficient early detection. Therefore, there is an urgent need for an automated and high-precision imaging analysis method to assist doctors in early diagnosis.

With the development of neuroimaging technology and deep learning, image analysis technology based on deep learning has been widely used in medical image diagnosis. These technologies can assist doctors in quickly and accurately diagnosing Parkinson's disease through the automated analysis of brain MRI, QSM, and other images, thereby providing early treatment opportunities, alleviating patients' symptoms, and improving their quality of life. The target detection algorithm based on deep learning not only has lower false detection and missed detection rates but also has stronger feature expression capabilities and higher detection accuracy, which is superior to traditional image detection algorithms. At present, there are two types of mainstream target detection algorithms: one is the two-stage detection algorithm represented by Faster R-CNN [4]; the other is the single-stage detection algorithm represented by the YOLO series [5–9]. The two-stage algorithm first generates candidate areas, and then classifies and locates these areas. The detection results are more accurate but the speed is relatively slow. The single-stage algorithm directly completes target detection through a single forward propagation process. It has a faster inference speed, but there may be certain challenges in small target detection and complex scenes.

Compared to other algorithms in the YOLO series, YOLOv5 has significantly improved performance, balancing detection accuracy and speed. Therefore, this article proposes an improved early diagnosis algorithm for Parkinson's disease using YOLOv5. The core innovation of this study lies in the triple upgrade of the YOLOv5 framework: firstly, the C3 module in the backbone is replaced with a CA attention mechanism [10], allowing the model to dynamically adjust attention based on local features of the image. Secondly, some convolutions in the Neck layer are replaced with full dimensional dynamic convolution modules [11] to enhance the model's image representation ability. Finally, replace the coupling head of the head layer with the decoupling head to improve the effectiveness of model detection. Through the synergistic effect of the above improvements, the model can not only efficiently identify small pathological changes, but also significantly reduce the rates of false positives and false negatives.

The experimental results show that the improved YOLOv5 algorithm performs better than other YOLO series algorithms in the diagnosis of Parkinson's disease, with higher accuracy and sensitivity in detecting early features of Parkinson's disease, especially in capturing subtle lesion features. Provided doctors with more reliable and effective diagnostic tools.

## Related works

In recent years, with the advancement of artificial inteIn recent years, with the advancement of artificial intelligence technology, various statistical models have been used to process high-dimensional neuroimaging data, resulting in computer-aided diagnosis (CAD) models, which provide powerful tools for intelligent diagnosis of neurological diseases. In research based on medical image processing and Parkinson's imaging, two main methods are used: one is traditional machine learning, which constructs high-precision PD classification and diagnosis models through feature extraction and selection; The second is deep learning, which adopts an end-to-end model to achieve efficient PD diagnosis and prediction, providing an automated solution for PD classification diagnosis.

In traditional machine learning methods, Cheng et al. [12] characterized N1 slices layer by layer, enhanced iron content contrast regions, and achieved accurate visualization of N1 signs in substantia nigra using quantitative magnetization maps and improved True SWI (tSWI) forms. By quantitatively evaluating the loss of N1 signs, it was found that the sensitivity and specificity of this feature in distinguishing between the normal control group and idiopathic Parkinson's disease (PD) reached 85% and 1%, respectively, highlighting the effectiveness of N1 markers in PD diagnosis. Anita et al. [13] proposed a three-dimensional brain functional imaging (SPECT) method for early diagnosis of Parkinson's disease. This method extracts shape and image surface-related features from three different sets of Volume Rendering Image (VRI) slices, uses genetic algorithms to select the optimal features in the feature set, and then uses an Extreme Learning Machine (ELM) classifier with radial basis function (RBF) kernel function for classification. The accuracy is as high as 99.12%, which is significantly better than support vector machines. Integrated learning technology has also been widely applied in the diagnosis of Parkinson's disease. Saleh et al. [14] proposed an integrated voting classifier that optimizes hyperparameters for prediction on two acoustic datasets of Parkinson's disease, with accuracy rates of 96.41% and 97.35%, respectively. This method performs particularly well in situations of limited and imbalanced data, demonstrating high accuracy and reliability. Ensemble learning has also demonstrated its superiority in other brain diseases, limited by the scarcity of annotated data and the complexity of images.

In deep learning-based methods, Zhu et al. [15] classified patients into five categories based on the severity of Parkinson's disease, and used deep learning neural networks and transfer learning based convolutional neural networks for model integration. The accuracy and recall of the hybrid model reached 0.94 and 0.95, respectively. Dhiman et al. [16] proposed a novel 2D convolutional neural network for learning complex patterns in MRI images to detect Parkinson's disease. Subsequently, transfer learning was applied through InceptionV3, and the overall accuracy of the model reached 95.29%. G. Mary et al. [17] used principal component analysis to perform feature selection on Parkinson's images and proposed a new classification model that achieves effective Parkinson's image classification by embedding Darknet 19 into CNN. In addition to Parkinson's disease, deep learning techniques have also shown great potential in the diagnosis of other diseases. Ullah et al. [18] proposed a fully automated brain tumor region segmentation technique based on multi-scale residual attention (MRA-UNET), which significantly improves the accuracy of brain tumor segmentation by using a multi-scale residual attention mechanism and adaptive regions of interest, especially in enhancing and processing core tumor regions. In addition, Ullah et al. [19] used the Multi-Task Semi-Supervised Adversarial Autocoding Framework (MTSS-AAE) to detect COVID-19 in another study. This framework effectively utilizes a large amount of unlabeled data by introducing auxiliary tasks such as pneumonia and pleural effusion detection, thereby significantly improving the accuracy of COVID-19 detection.

Future research can refer to these methods when processing Parkinson's disease (PD) data, and improve the accuracy and comprehensiveness of diagnosis by combining brain image segmentation techniques and comprehensive analysis of multimodal data (such as MRI, PET, QSM, etc.). Introducing auxiliary tasks such as age, gender, and other neurodegenerative diseases will help identify more subtle pathological features, enhance the sensitivity and specificity of early diagnosis, and provide patients with more accurate and comprehensive diagnostic tools.

## Related technologies

### MRI technology

Magnetic resonance imaging (MRI) is a non-invasive medical imaging technique that provides high-resolution medical images by reconstructing the signals generated by atomic nuclei resonating in a magnetic field [20].

The main imaging sequences of MRI include T1WI, T2WI, and T2 weighted imaging fluid-attenuated inversion recovery (T2WI-FLAIR), which can reveal changes in the structure and function of the PD brain, capture the absence of dopaminergic neurons, and α- The aggregation of synaptic nucleoproteins and other neuropathological markers changes [21], forming specific texture patterns. By utilizing artificial intelligence technology, these texture features can capture subtle information in medical images that is difficult to evaluate with the naked eye, overcoming the limitations of visual diagnosis and providing assistance for the diagnosis and differentiation of Parkinson's disease.

### YOLOv5

The basic framework of YOLOv5 consists of four parts, namely Input, Backbone layer, Neck layer, and Prediction. The Input module adopts the Mosaic data enhancement strategy to expand the dataset through random scaling, random cropping, and random arrangement. The Backbone layer uses the CSPDarknet53 framework and contains three parts: Conv, C3, and SPPF. Among them, the Conv and C3 layers enhance feature extraction through connections, and the C3 module divides features of different sizes into two parts, one part is passed downward, and the other part is integrated with other features through the Neck layer, effectively avoiding the phenomenon of gradient disappearance [22]. Through three maximum feature pooling layers, the SPPF structure fuses feature maps with different receptive fields and, at the same time, enriches the expressive ability of feature maps and further improves the running speed. The Neck part utilizes a feature pyramid network (FPN) and path aggregation network (PANet) to integrate the image features at this stage [23]. FPN structure fuses semantic feature information in a top-down path and PANet fuses features in a bottom-up manner to enhance positioning capabilities at multiple scales. Finally, the Prediction module performs target prediction and adopts the non-maximum suppression (NMS) algorithm to remove bounding boxes with more overlaps and generate the final detection result [24].

Compared with other YOLOv5 models, YOLOv5 has been widely validated and applied in the community, demonstrating mature and stable performance, making it particularly suitable for handling complex medical image analysis tasks. Thanks to the active open-source community support, YOLOv5 provides rich pre-trained models and technical support, providing a reliable foundation and resources for research and application development.YOLOv5s has a more streamlined network structure and parameter quantity while demonstrating superior detection performance, making it particularly suitable for early Parkinson's disease diagnosis and prediction that requires high accuracy. Therefore, this article chooses YOLOv5s as the basic model for Parkinson's image detection.

## Methodology

Based on the particularity of medical images, MRI images contain many tiny abnormal areas and texture details, which require stronger feature expression for detection and classification.

**Fig. 1:**
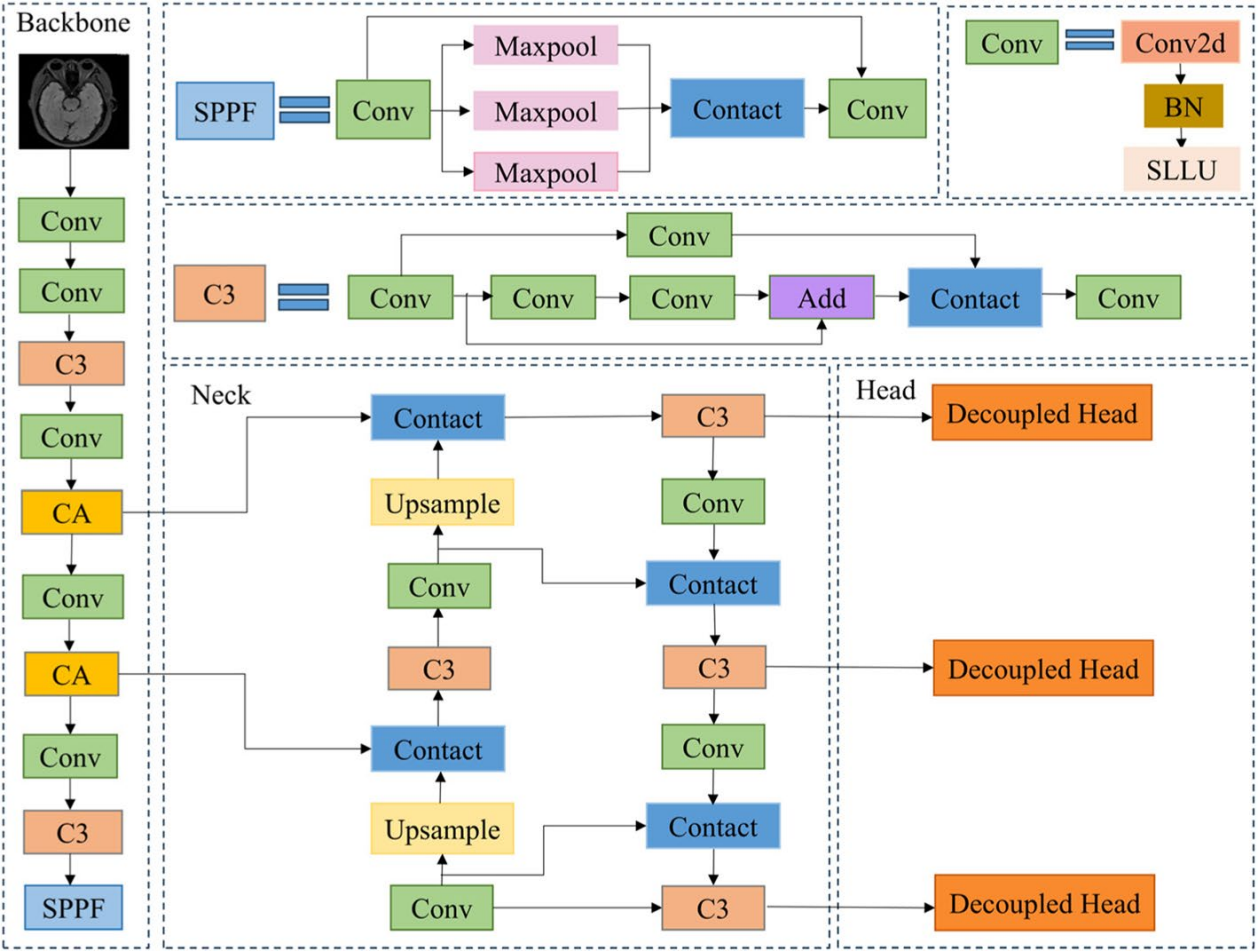
Improved YOLOv5 network structure

As shown in the network structure diagram of Fig. 1, this paper takes the YOLOv5s network as the basic framework to improve it. Aiming at the uncertainty of Parkinson's disease areas, a CA position attention mechanism is introduced, and the C3 module in the Backbone is replaced by a CA module so that the model can dynamically adjust the attention according to the local features of images and focus more on potential disease areas. To increase the sensitivity of feature extraction, partial convolutions in the Neck layer are replaced with full-dimensional dynamic convolution modules, which helps the model to extract Parkinson's disease features more accurately and enhance the learning and expression capabilities of the network. In response to the high requirements for Parkinson image detection accuracy, the coupling head of the Head layer is replaced with a decoupling head to decouple the classification task from regression, to improve the effect of model detection [25].

### Improvement of Backbone layer

During Parkinson's image detection, to concentrate the model's attention on the lesion area of the MRI image, we pay attention to the subtle changes in the Parkinson image. This paper adds an attention module into Backbone to enhance the feature extraction capabilities of the model for Parkinson's disease areas.

The attention mechanism allows the model to focus on key information, strengthen the weight of beneficial features, and inhibit redundant features [26]. Traditional SENet (squeeze-and-excitation networks) mainly looks into inter-channel information and ignore the importance of position information. CBAM (Channel and Spatial Attention) combines spatial and channel information but only considers information within the range of local spatial locations. On this basis, Ullah et al. [27] proposed a dense attention mechanism network that combines dense layers, channel attention layers, adaptive downsampling layers, and label smoothing regularization loss functions. More refined processing of image features has improved the model's ability to distinguish COVID-19 infected areas in complex backgrounds. In the image detection scenario of Parkinson's disease, the CA attention mechanism can embed positional information into channel attention, considering the relationships between different channels and the long-term dependencies between spatial positions at the top of the window [28].This fine attention mechanism can more accurately locate the possible lesion areas of the patient, and improve the network's perception ability for small or fuzzy abnormal signals. Therefore, this article chooses to incorporate the CA attention mechanism.

The coding of the CA module is divided into two steps: coordinate information embedding and coordinate attention generation. Its specific structure is as follows.

**Fig. 2:**
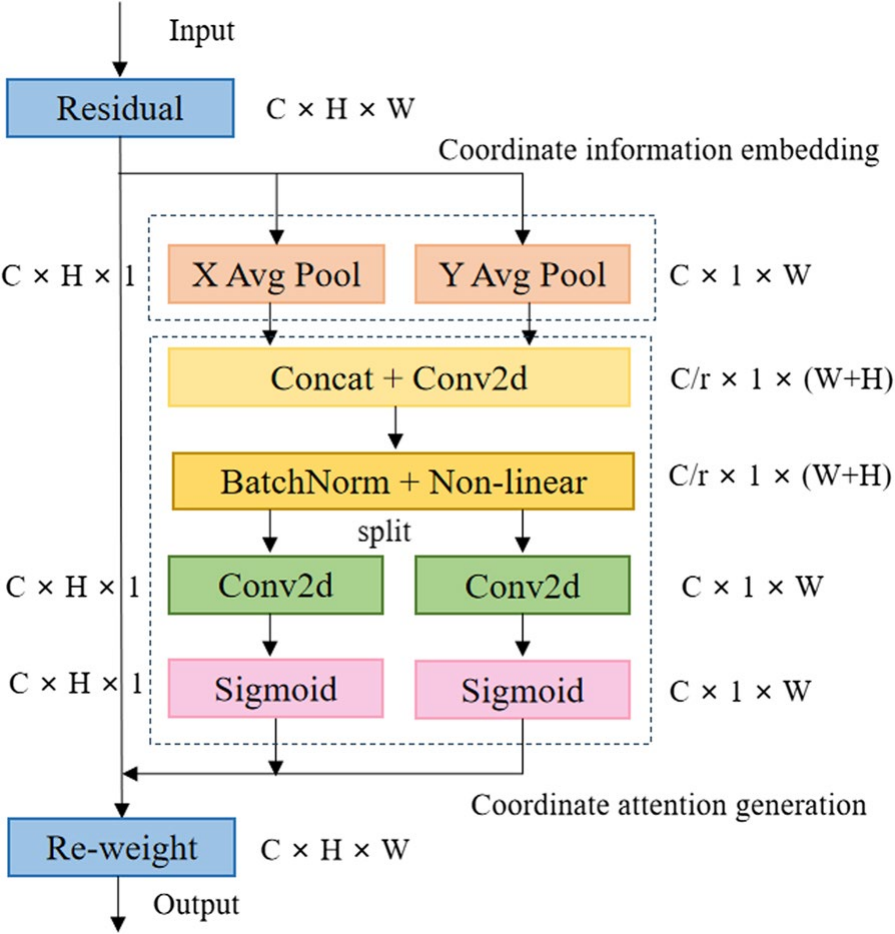
CA module structure diagram

As shown in Fig. 2, The CA attention mechanism decomposes global pooling into a pair of one-dimensional feature encodings. For the input features$\text{X}$, the module takes two sets of pooling kernels $(\text{ H }, 1 )$ and $(1,\text{W})$ to encode each channel along the horizontal X and vertical Y directions. The output with height of $\text{h}$ and width of $\text{w}$ on the channel $\text{c}$ are as described by (1) and (2), respectively.

1 $${\text{z}}_{\text{c}}^{\text{h}}\left(\text{h}\right)=\frac{1}{\text{W}}\sum_{0\le \text{i}\le \text{W}}{\text{x}}_{\text{c}}\left(\text{h},\text{i}\right)$$

2 $${\text{z}}_{\text{c}}^{\text{w}}\left(\text{w}\right)=\frac{1}{\text{H}}\sum_{0\le \text{j}\le \text{H}}{\text{x}}_{\text{c}}\left(\text{j},\text{w}\right)$$

The feature maps generated by formulas (1) and (2) are spliced together, and a shared 1 × 1 convolution is used for feature transformation. The final output result $\text{f}$ is expressed as follows:

3 $$\begin{array}{c}f=\delta \left({\text{F}}_{1}\left(\left[{\text{z}}^{\text{h}},{\text{z}}^{\text{w}}\right]\right)\right)\end{array}$$

where, $\updelta$ represents a nonlinear activation function, $\text{f}$ is the intermediate feature mapping that encodes the spatial information in the horizontal and vertical directions.

Divide the $\text{f}$ into two tensors ${\text{f}}^{\text{h}}\epsilon {\text{R}}^{\text{C}/\text{r}\times \text{H}}$ and ${\text{f}}^{\text{w}}\epsilon {\text{R}}^{\text{C}/\text{r}\times \text{W}}$ along the spatial dimension and use two 1 × 1 convolution ${\text{F}}_{\text{h}}$ and ${\text{F}}_{\text{w}}$ to transform ${\text{f}}^{\text{h}}$ and ${\text{f}}^{\text{w}}$ to the same number of channels as the input $\text{X}$. The calculation process is expressed in (4) and (5), respectively.

4 $$\begin{array}{c}{\text{g}}^{\text{h}}=\sigma \left({\text{F}}_{\text{h}}\left({\text{f}}^{\text{h}}\right)\right)\end{array}$$

5 $$\begin{array}{c}{\text{g}}^{\text{w}}=\sigma \left({\text{F}}_{\text{w}}\left({\text{f}}^{\text{w}}\right)\right)\end{array}$$

where, $\upsigma$ is the sigmoid activation function. Finally, the output of coordinate attention block ${\text{y}}_{\text{c}}\left(\text{i},\text{j}\right)$ is expressed as:

6 $$\begin{array}{c}{\text{y}}_{\text{c}}\left(\text{i},\text{j}\right)={\text{x}}_{\text{c}}\left(\text{i},\text{j}\right)\times {\text{g}}_{\text{c}}^{\text{h}}\left(\text{i}\right)\times {\text{g}}_{\text{c}}^{\text{w}}\left(\text{j}\right)\end{array}$$

**Fig. 3:**
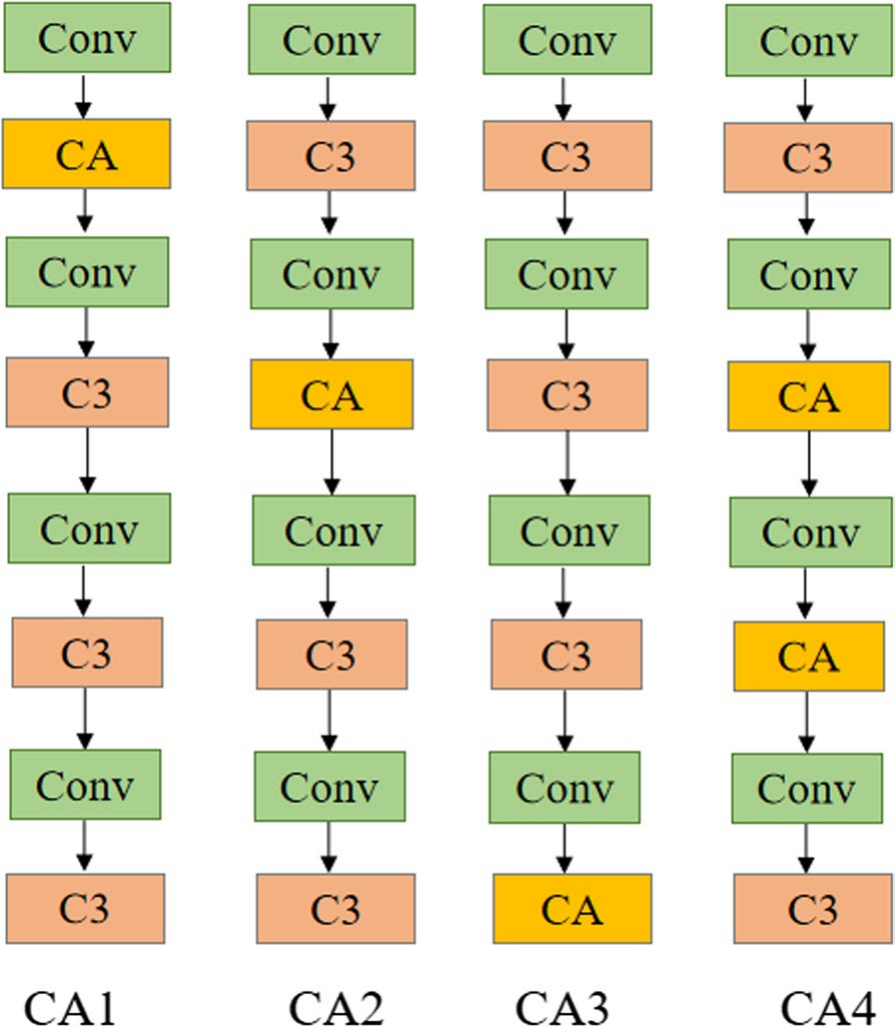
CA module location

This paper replaces the C3 module in Backbone with the CA attention module. During the feature extraction process, the introduced CA attention module adaptively adjusts the weights in the feature map, allowing the model to focus more on the small and weak lesion features in the MRI image, thereby improving the model's ability to perceive targets. The CA attention module enhances the interaction between channels and spaces by introducing coordinate information, enabling the model to more accurately locate and identify weak lesion areas in MRI images of Parkinson's disease.This paper was designed to insert CA modules at four different positions, as shown in Fig. 3, and the specific effects were verified through experiments.

### Improvements of Neck layer

The difference between the brain images of Parkinson's patients and healthy people is very small, so it is difficult for ordinary convolution to fully extract distinguishing features. Therefore, this paper introduces full-dimensional dynamic convolution in the Neck layer to replace ordinary convolution for feature extraction.

Generally, traditional dynamic convolution only achieves dynamics in the dimension of the number of convolution kernels, that is, weigh multiple convolution kernels to adapt to different input features. The full-dimensional dynamic convolution ODConv utilizes multi-dimensional attention mechanism to learn four types of convolution kernel attention along the four dimensions of kernel space (space kernel size, number of input channels, number of output channels, number of convolution kernels) in a parallel manner attention [29]. The following figure is the operation process of the ODConv module.

**Fig. 4:**
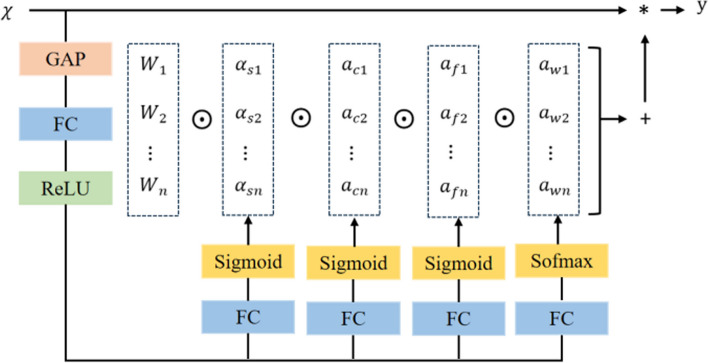
ODConv module operation process

As can be seen from Fig. 4, $\text{x}$ after the input passes through the global average pooling (GPA), fully connected (FC) and ReLU activation function, 4 head branches are established and 4 types of attention scalars of ODConv convolution module. Each branch passes through FC layers with sizes of $\text{k}\times {\text{k}}_{\backprime}{\text{c}}_{\text{in}}\times {1}_{\backprime}{\text{c}}_{\text{out}}\times 1$ and$\text{n}\times 1$, respectively. Among them, the first, second, and third branches generate normalized attention scalars $\alpha_{si}\backprime\ \alpha_{ci}{\backprime\ }\ \mathrm{a}_{fi}$ from the feature variables output by the FC layer through the Sigmod activation function. The fourth branch produces a normalized attention scalar ${\alpha }_{\text{wi}}$ through Sofmax activation function [21]. The calculation formula of ODconv is shown in (7).

7 $$\begin{array}{c}{y}=( {{\alpha }}_{\upomega 1}\odot {{\alpha }}_{{f}1}\odot {{\alpha }}_{{c}1}\odot {{\alpha }}_{{s}1}\odot {{W}}_{1}+\dots \\ +{{\alpha }}_{{\omega n}}\odot {{\alpha }}_{{fn}}\odot {{\alpha }}_{{cn}}\odot {{\alpha }}_{{sn}}\odot {{W}}_{{n}})*\mathrm{x}\end{array}$$

where, ${\alpha }_{{\omega i}}\in {R}$ represents the attention scalar of convolution kernel ${\text{W}}_{\text{i}}$,${{\alpha }}_{{si}}\in {{{R}}^{{k}\times {k}}}_{\backprime}{{\alpha }}_{{fi}}\in {{R}}^{{{c}}_{{out}}}$ and ${{\alpha }}_{{ci}}\in {{R}}^{{{c}}_{{in}}}$ denotes the newly introduced three dimensions of attention, which are calculate along the spatial dimension of convolution kernel ${\text{W}}_{\text{i}}$, the input channel dimension and the output channel dimension, respectively. $\odot$ describes the multiplications along different dimensions of convolution kernel. $\text{x}$ is input features.

**Fig. 5:**
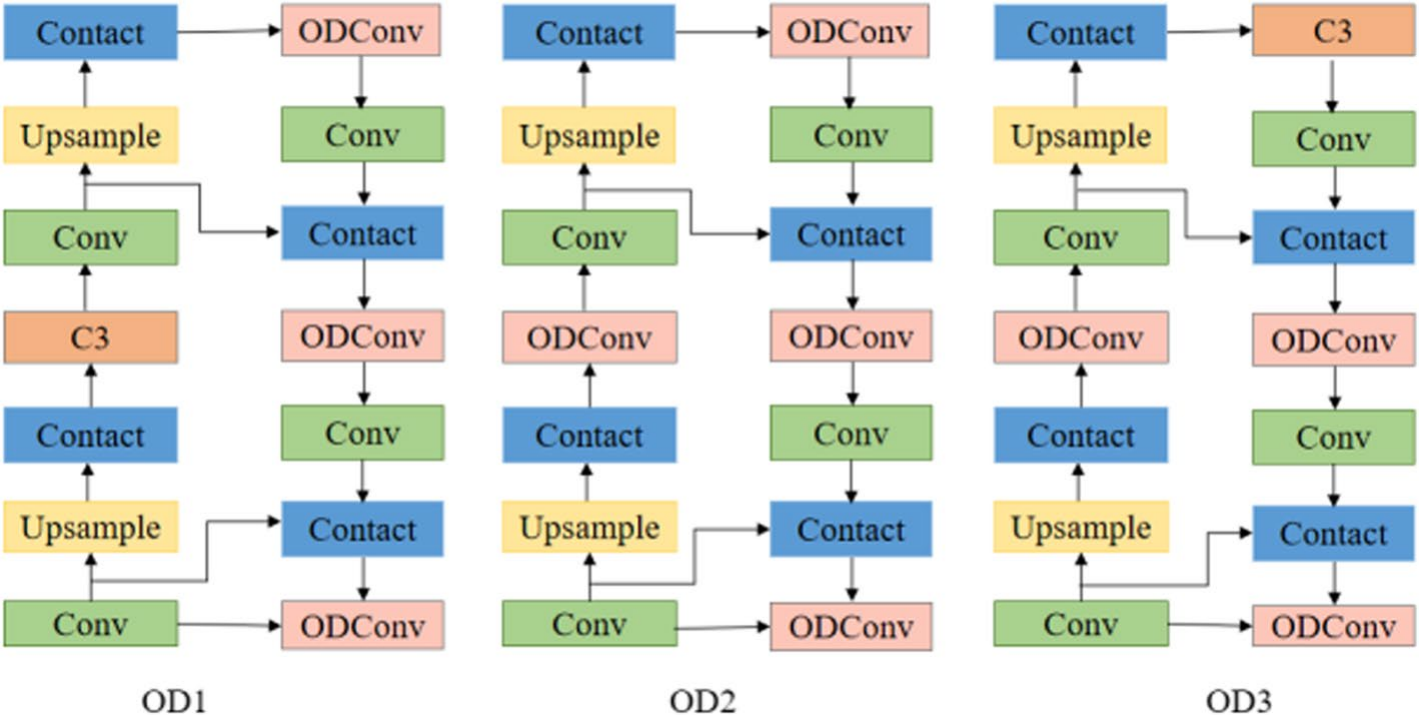
ODConv module location

This paper replaces the C3 module in the Neck layer with ODConv dynamic full-dimensional convolution and weights the convolution kernel from four dimensions, to enhance the feature extraction capability of the network and effectively improve the accuracy of the model. As shown in Fig. 5, three different embedding methods are designed and their specific effects are verified through experiments.

### Improvement of Head detection layer

**Fig. 6:**
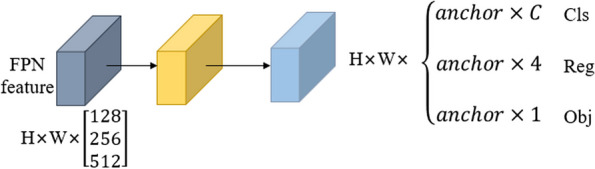
YOLOv5 coupling head structure

YOLOv5 detection adopts the coupling head, and through a series of convolution and fully connected layers, it can simultaneously predict category labels (Cls), prediction frame coordinates (Reg), and target confidence (Obj) at different scales. However, in Parkinson's image detection, there is a conflict between the classification task and the regression task. As shown in Fig. 6, the detection head of YOLOv5 combines classification and regression tasks for calculation. This coupled prediction head usually has the problem of inaccurate positioning.

To solve the conflict between Parkinson's MRI image classification and regression task, the convergence of the model is accelerated and the overall performance of the model is improved. In this paper, the decoupled prediction head (Decoupled head) of YOLOX [30] is applied to calculate the classification task and the regression task separately.

**Fig. 7:**
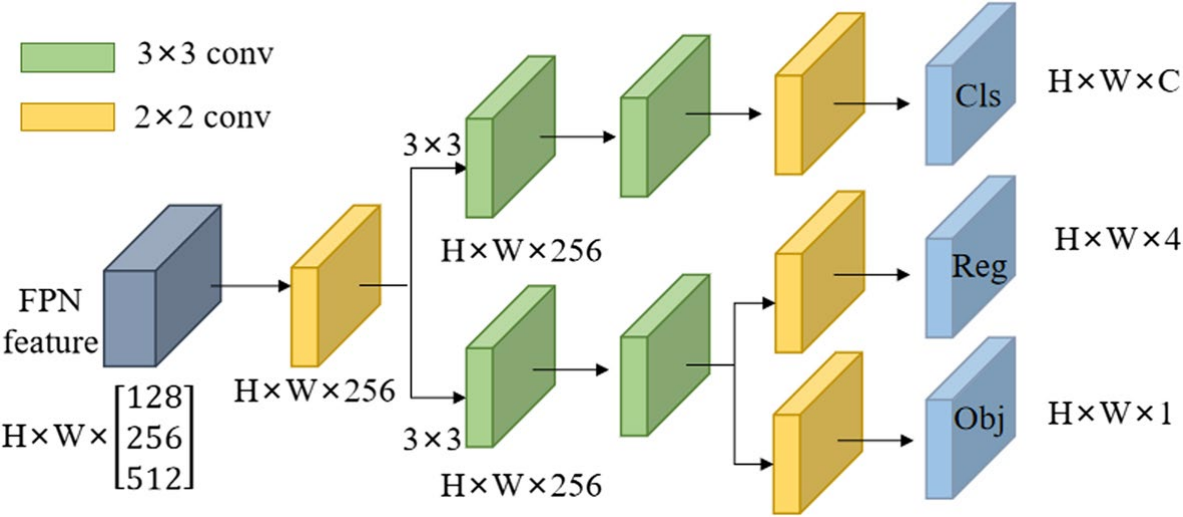
Improved decoupling head structure

As shown in Fig. 7, the input of the decoupled head is treated with dimensionality reduction through a 1 × 1 convolutional layer. Then, it goes through two branches, one for the classification task of Parkinson's MRI images, and the other for the regression task of MRI image detection. There are two 3 × 3 convolutional layers on each branch. The regression task is divided into two branches, which are responsible for the regression tasks of location and confidence of Parkinson's image detection, respectively [31]. By replacing the detection head, the network can better handle the conflict between classification and regression tasks, thereby improving the accuracy of model detection.

## Experimental evaluation

### Experimental data source

The data used in this experiment was collected from real patients at Ningxia Medical University. The project data of Ningxia Medical University is based on the Ningxia Key Laboratory of Brain Diseases, and the dataset is collected by an intelligent 3.0 T high-end magnetic resonance imaging system (MRI). In terms of ethical issues in medical data processing, this study adopted data encryption and anonymization to ensure the protection of patient privacy and prevent identity leakage. In the future, when collecting data from different institutions, we plan to draw on the federated learning technology of Faizan et al. [32] to curb data leakage incidents and prohibit unauthorized data access, building a solid defense line for data security.

In addition, patients have clear informed consent before participating in the study, including the collection, storage, and processing of data. They can choose to withdraw from the study at any time, and the data is only used for scientific research purposes. This experiment works closely with the ethics committee to ensure that the research design and implementation comply with ethical standards and adhere to best practice principles. Through regular education and training, enhance the awareness and ability of medical staff and researchers to address ethical issues, and ensure strict compliance and implementation of ethical standards and legal requirements during the research process.

**Fig. 8:**
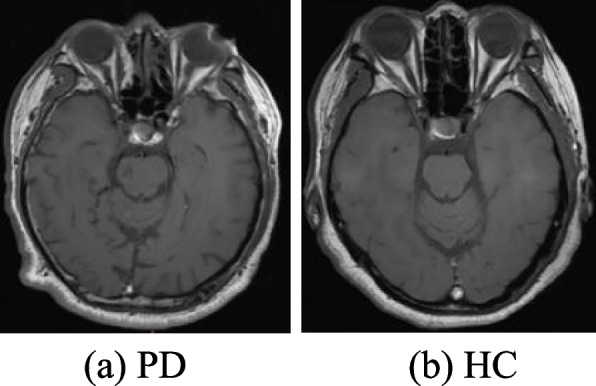
Parkinson's MRI images

The original dataset contains 582 images from 50 healthy individuals (HC) and 54 Parkinson's disease patients (PD).Fig. 8 shows sample images of HC and PD groups.

All datasets in this experiment were manually labeled using Labelme software, and Parkinson's images were divided into two types of labels: HC and PD. Randomly shuffle the annotated dataset and divide it into an 80% training set and a 20% testing set. All models are trained and tested on the same computer.The experimental dataset information is shown in Table 1.

**Table 1:** Subject information

Group	Number of people	Gender (male /female)	Age range	Average age	Number of images
HC	50	24/26	46 ~ 70	57	281
PD	54	29/25	48 ~ 78	60	301

### Experimental environment

The experimental environment GPU for this article is RTX 3090 (24 GB). The CPU is 16 vCPU Intel (R) Xeon (R) Platinum 8350C CPU @ 2.60 GHz. Using Python 3.8.10 version, the deep learning framework is based on PyTorch 1.11.0, and Cuda version is 11.3.

During the model training process, the SGD optimizer is used to optimize the network, with a learning rate of 0.001 and cosine annealing used to adjust the learning rate. The total number of epochs is set to 300.

#### Evaluating indicator

The experiment used accuracy P (Precision), recall R (Recall), and mean average precision as evaluation indicators for the Parkinson's MRI image diagnostic task. The corresponding calculation formula is as follows:

8 $$\text{P}=\frac{TP}{TP+FP}$$

9 $$\text{R}=\frac{TP}{TP+FN}$$

10 $$\text{AP}={\int }_{1}^{0}P(R)dR$$

11 $$\text{MAP}= \frac{1}{n}\sum_{i=1}^{n}A{P}_{i}$$

Among them, TP is the number of correctly assigned positive samples, FP is the number of incorrectly assigned positive samples, FN is the number of incorrectly assigned negative samples, and N is the total number of categories. AP measures the recognition accuracy of the model in each category, while mAP measures the average recognition accuracy of the model in all categories. The AP and mAP at an IOU threshold of 0.5 are used in the article to measure the quality of the model.

### Experimental result

#### The impact of CA attention mechanism insertion position on the network

To verify the effectiveness of the CA attention module in Parkinson's image detection and investigate the impact of embedding attention mechanisms in Backbone on algorithm performance, this paper conducted experiments with four different embedding methods. The embedding position of the CA attention module is shown in Fig. 3, and the experimental results are shown in Table 2.

**Table 2:** Comparison of CA at different network layers

Model	P%	R%	mAP%	GFLOPs	Parameter
CA1	91.9	90.1	97.5	14.8	6998583
CA2	94.6	97.6	**98.7**	14.5	6907032
CA3	94.0	94.5	97.6	14.8	**5859983**
CA4	**95.0**	**97.9**	97.7	**12.4**	6289831
YOLOv5s	94.2	94.9	97.7	15.8	7015519

The results showed that the insertion position of attention in CA1 and CA3 slightly decreased the detection performance of the model, while the embedding method of CA2 and CA4 improved the accuracy of the model. Among them, CA4 has the best effect, with an accuracy and recall improvement of 0.8% and 3.0%, respectively, compared to the original model. Analysis shows that in CA1 and CA3, embedding attention at the front and end of the backbone layer can affect the transmission and extraction of image information, resulting in the model being unable to fully learn effective features of Parkinson's disease. CA2 and CA4 focus their attention on the middle part of the backbone layer, better balancing the extraction of low-level and high-level features, thereby maintaining the stability of the network structure and effective transmission of features. Therefore, this experiment chooses the fourth CA attention replacement method as the improvement strategy for the backbone layer.

#### The impact of ODConv insertion position on the network

To verify the impact of ODConv full-dimensional dynamic convolution modules at different positions on the improved algorithm, this paper conducted experiments on three different embedding methods, and their embedding positions are shown in Fig. 5. The specific results are shown in Table 3.

**Table 3:** Comparison of ODConv at different network layers

Model	P%	R%	mAP%	GFLOPs	Parameter
OD1	96.0	**96.2**	98.4	4.1	**2527366**
OD2	94.5	94.2	98.4	4.1	2674435
OD3	**96.1**	94.4	**98.8**	**3.3**	3831002
YOLOv5s	94.2	94.9	97.7	15.8	7015519

The experimental results show that the placement of ODConv in all three positions improves the performance of the model. Among them, OD3 has the highest accuracy, with an accuracy rate and average accuracy value that are 1.9% and 1.1% higher than the original model, respectively. The GFLOPs and parameter count of the OD3 model are relatively low, which can achieve object detection tasks at a lower computational cost while maintaining performance.

#### Ablation experiment

To verify the effectiveness of the algorithm proposed in this paper and explore the impact of each module on network performance, ablation experiments were conducted in this paper. The experiment used the same dataset, with YOLOv5s as the base network, and added the CA attention module, ODConv dynamic convolution, and decoupling head one by one. A total of six experiments were designed, and the experimental results are shown in Table 4.

**Table 4:** Results of ablation experiment

CA	OD	Decoupled head	P%	R%	mAP@0.5%
×	×	×	94.2	94.9	97.7
√	×	×	95.0	**97.9**	97.7
×	√	×	96.1	94.4	**98.8**
×	×	√	95.8	92.8	98.4
√	√	×	95.3	97.3	97.8
√	√	√	**96.1**	97.4	98.6

From the experimental results in Table 4, it can be seen that after adding the CA attention module to the Backbone layer, the accuracy and recall of the model increased by 0.8% and 3.0%, respectively, indicating that the attention mechanism can focus more on the lesion features of Parkinson's MRI images, effectively improving the accuracy of Parkinson's prediction. After adding OD dynamic convolution to the Neck layer, the model accuracy is slightly improved. After decoupling the Head layer, the average accuracy improved by 0.8%, indicating that the decoupling head helps to collaborate between classification and regression tasks, improving the accuracy of object detection. In addition, the introduction of lightweight CA attention modules and OD convolution modules can alleviate the problem of parameter increase caused by the addition of decoupling heads in the model. In the end, the improved model increased accuracy, recall, and average accuracy by 1.9%, 2.5%, and 0.9%, respectively, compared to the original model. From the results of the ablation experiment, it can be seen that the three modules have effectively improved the detection performance of Parkinson's images.

#### Model comparison experiment

To verify the effectiveness of this algorithm, other mainstream image detection algorithms Faster R-CNN [4], RetinaNet [33], SSD [34], EfficientNet [35], YOLOv3 [7], YOLO7 [9], and YOLOv8n were used for comparative experiments on the same Parkinson's MRI dataset. Precision, Recall, and mAP were used for evaluation and comparison, and the comparative experimental results are shown in Table 5.

**Table 5:** Comparative experimental results

Model	P%	R%	mAP@0.5%
Faster R-CNN [4]	64.7	76.4	75.6
RetinaNet [33]	66.1	69.2	74.7
SSD [34]	67.0	56.9	71.4
EfficientNet [35]	90.1	89.6	98.1
YOLOv3 [7]	64.5	93.6	81.0
YOLOv7 [9]	85.4	93.7	94.4
YOLOv8n	84.7	89.1	92.5
YOLOv5s	94.2	94.9	97.7
Ours	**96.1**	**97.4**	**98.6**

From the table, it can be seen that the improved YOLOv5 algorithm has the highest detection accuracy among all algorithms, with an average accuracy of at least 20 percentage points higher than the Faster R-CNN, RetinaNet, and SSD algorithms. It can be seen that these three algorithms have poor detection and classification capabilities for Parkinson's MRI images and are not suitable for Parkinson's image detection tasks.In contrast, the YOLO series algorithms have shown high accuracy, with an average accuracy of over 90% for YOLOv7 and YOLOv8, but their accuracy has decreased by 8.8% and 9.5% respectively compared to YOLOv5. EfficientNet achieved 98.1% in the mAP metric, which is only 0.5 percentage lower than the improved algorithm in this paper. It is also better than other YOLO series algorithms in terms of accuracy and recall, but still lower than the YOLOv5 algorithm.The improved algorithm in this article has increased accuracy, recall, and average accuracy by 1.9%, 2.5%, and 0.9% compared to the original YOLOv5 algorithm. In summary, the improved algorithm proposed in this article demonstrates significant advantages in the field of Parkinson's disease MRI image detection compared to a series of mainstream algorithms including YOLOv5 original and other advanced algorithms.

#### Comparison experiment of model detection effect

**Fig. 9:**
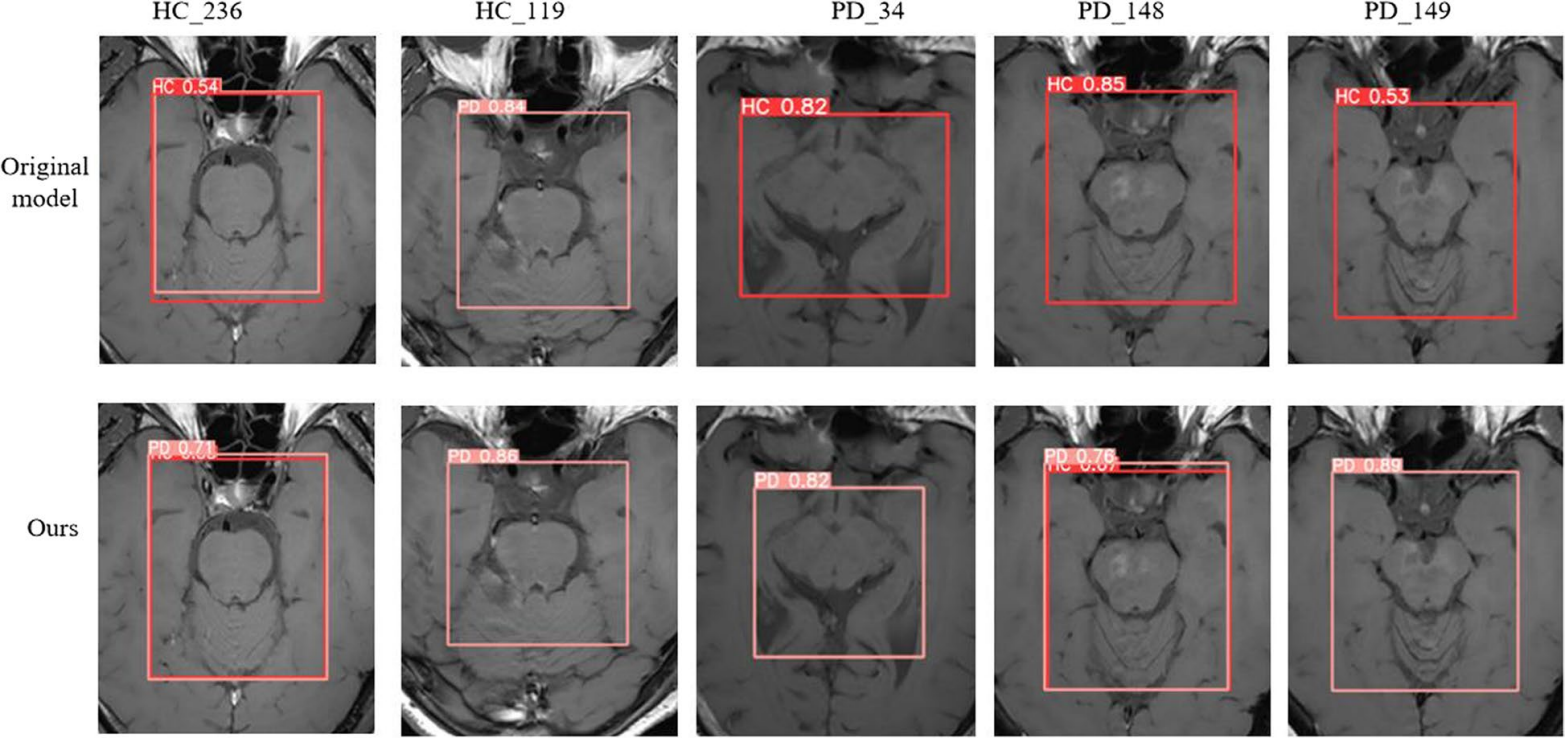
Comparison chart of detection results

To compare the detection and classification performance of the original model and the improved model on Parkinson's MRI images more intuitively, this paper selected 53 Parkinson's images for testing, and the partial detection results are shown in Fig. 9.

Among the 53 test images, The original model experienced 9 detection errors, including 6 false positives and 3 misclassifications of healthy individuals and patients.

**Fig. 10:**
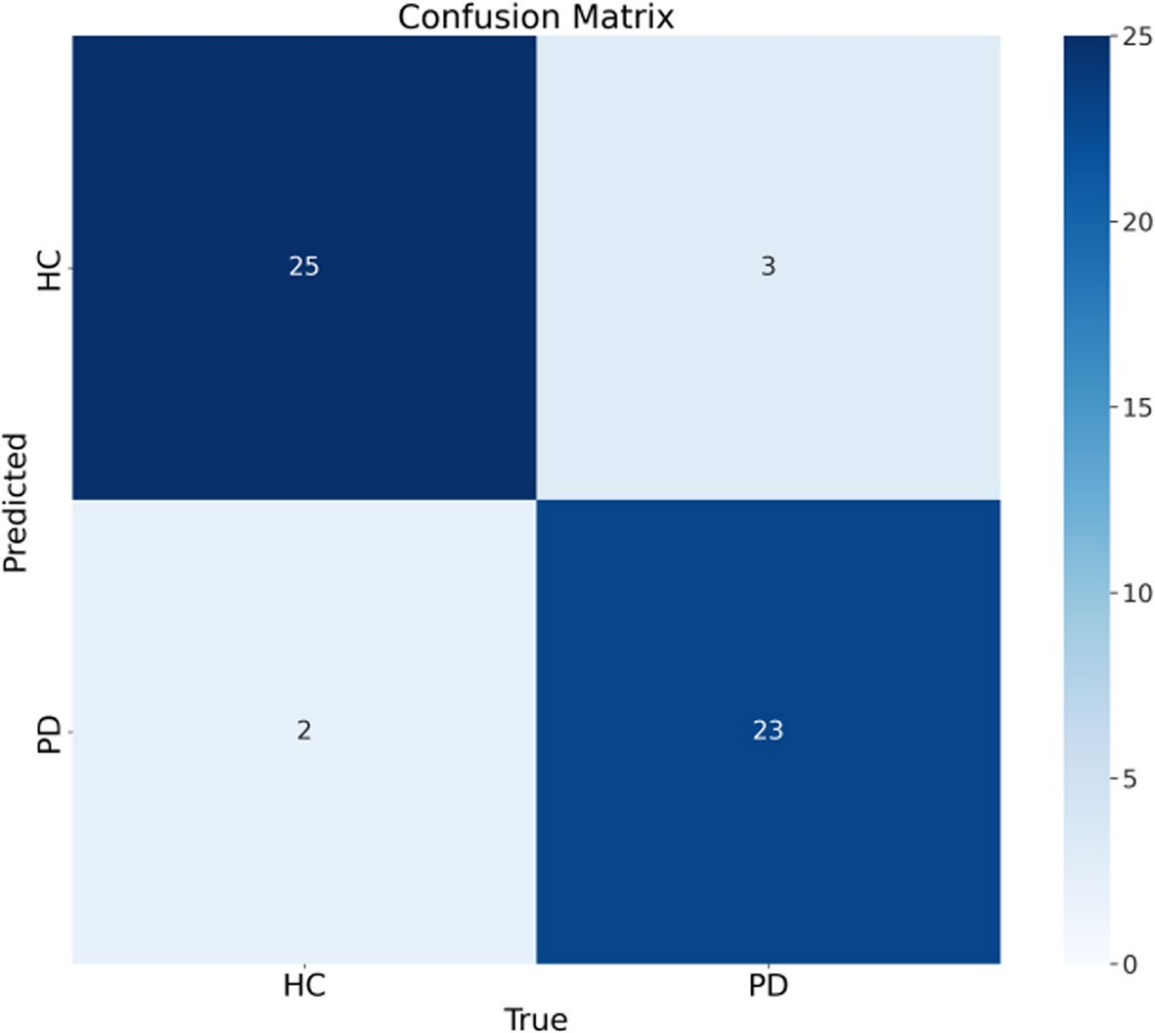
Confusion matrix

By contrast, it can be seen from the confusion matrix in Fig. 10 that the improved model only detects errors five times. From the figure, it can be seen that three of the images (HC_119, HC_236, and PD_148) have detection errors in both the original model and the improved model. Overall, out of the 5 detection issues identified in the improved model, two were detection errors, and the other three were situations where healthy individuals and patients were correctly detected. Therefore, compared to the original model, the improved model has a higher detection accuracy and is more suitable for the detection task of Parkinson's MRI images.

**Fig.11:**
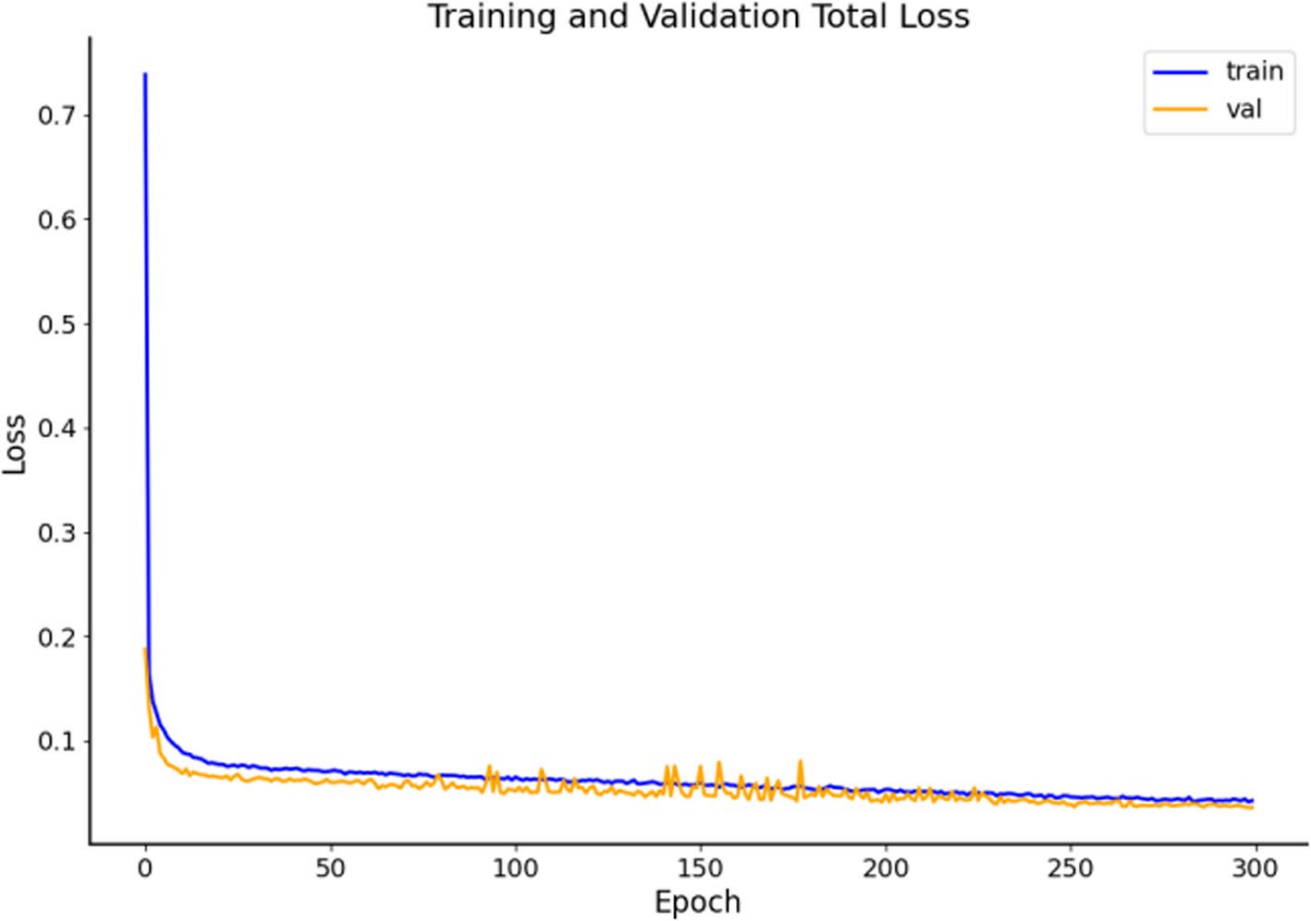
Model loss curve

In addition, from the training loss curve in Fig. 11, it can be seen that the improved model converges rapidly during the training process, and the training and validation losses are significantly reduced and tend to stabilize. This indicates that the improved model has good stability and effectiveness in the training process of Parkinson's diagnosis tasks, and can effectively cope with Parkinson's image detection tasks.

## Discussion and challenges

The Parkinson's dataset used in this study included 104 participants with an age range of 46 to 78 years old. Although the dataset covers a wide age range, which helps the model train in different age groups, the number of participants is limited and there is a lack of racial diversity statistics, which may limit the model's adaptability in a wider population. In the face of data scarcity and variable quality, consider using transfer learning [36] to effectively learn and recognize image features related to Parkinson's disease. In the future, when collecting more datasets, it is planned to adopt a lightweight integrated model [37] to reduce the computational complexity of the model and improve its flexibility and scalability in handling diverse and complex image data.

In practical applications, the performance of the improved YOLOv5 algorithm under different conditions needs further discussion. Firstly, the imaging parameters and quality of different MRI machines may affect the extraction of image features. Therefore, this study adopted the DICOM standard to unify data formats and plans to develop algorithms that adapt to different device parameters, design effective data integration solutions, and ensure that the model can be seamlessly integrated into existing medical systems. At the same time, technical support and training will be provided to ensure that medical personnel can correctly use and understand the output of the model, thereby promoting the successful application of the improved YOLOv5 model in diverse medical environments.

In terms of efficiency and accuracy, the core of this study is to improve the accuracy of early diagnosis of Parkinson's disease. Due to the complex convolution operations and attention mechanism required by the YOLOv5 algorithm, it may result in slower inference speed on slower hardware platforms. At the same time, the performance of the model in handling extreme or abnormal situations, as well as fault points such as differences in image quality in the dataset, may challenge diagnostic accuracy. When weighing accuracy and efficiency, the diagnosis of Parkinson's disease requires highly reliable results, and this study focuses on accuracy to ensure that patients receive timely treatment and management.

## Conclusion

This paper takes Parkinson's MRI images as the research object and proposes an improved YOLOv5s Parkinson image detection algorithm to solve the problems of low Parkinson recognition rate and high early diagnosis delay rate. This paper first introduces the CA attention module into Backbone to improve the model's attention to Parkinson's fuzzy lesion areas. Subsequently, full-dimensional dynamic convolution is inserted into the Neck layer to enhance feature extraction and obtain richer feature information. Finally, the detection head of Head layer is replaced with the decoupled head, which reduces the coupling of the model and improves the detection accuracy and robustness of the model. Experimental results indicate that the improved algorithm in this paper has a precision rate of 96.1%, a recall rate of 97.4%, and a mAP of 98.6%. It can accurately detect and identify complex Parkinson's MRI images and provide clinical assistance for early diagnosis.

Future research will further enhance the effectiveness of early diagnosis and management of Parkinson's disease. In terms of the Internet of Things, real-time monitoring and data transmission will be achieved through intelligent sensors and wearable devices, supporting remote medical care and real-time intervention, improving the timeliness and coverage of diagnosis. In the field of artificial intelligence, we will explore multimodal data fusion and personalized treatment plans, using deep learning models to analyze MRI images, gene data, and clinical records, providing more accurate diagnosis and personalized treatment. In terms of model improvement, the YOLOv5 model will be further optimized and improved, exploring more effective model pruning and fusion methods to enhance model performance. In addition, we will also consider applying the improved model to other medical image diagnosis tasks and comparing it with other deep learning models to better apply it in clinical practice.

## Data Availability

The datasets generated and analyzed during the current study are not publicly available due to the limitations in hospital confdentiality agreements, but they are available from the corresponding author upon reasonable request.
